# Many Infants and Young Children Are Not Compliant with Mexican and International Complementary Feeding Recommendations for Milk and Other Beverages

**DOI:** 10.3390/nu10040466

**Published:** 2018-04-10

**Authors:** Myriam C. Afeiche, Salvador Villalpando-Carrión, Kathleen C. Reidy, Lisa R. Fries, Alison L. Eldridge

**Affiliations:** 1 Institute of Nutritional Science, Nestlé Research Center, Route du Jorat 57, 1000 Lausanne, Switzerland; Myriam.AfeicheZehil@rd.nestle.com; 2 Hospital Infantil de Mexico, Gastroenterology and Nutrition, Mexico City and Nestlé Infant Nutrition, Av. Ejercito Nacional 453, Delegacion Miguel Hidalgo, Col. Granada, Mexico City 11520, Mexico; Salvador.Villalpando@MX.nestle.com; 3 Nestlé Nutrition Global R&D, 12 Vreeland Road, Florham Park, NJ 07932, USA; Kathleen.Reidy@rd.nestle.com; 4 Consumer Science and Applied Nutrition, Institute of Material Science, Nestlé Research Center, Route du Jorat 57, 1000 Lausanne, Switzerland; Lisa.Fries@rdls.nestle.com

**Keywords:** ENSANUT 2012, infant diet, young child diet, milk intake, beverages intake

## Abstract

Mexican and international authorities provide guidelines for milk and beverage consumption for young children. This study classifies beverages as appropriate or inappropriate by age (0–5.9, 6–11.9, and 12–23.9 months) and details consumption patterns, amounts consumed, and the associated socio-demographic characteristics. Analysis of the Mexican National Nutrition and Health Survey (ENSANUT 2012) was conducted (*n* = 949). Among 0–5.9 month olds, 66.7% consumed either breast milk, infant formula, or a combination with no other beverages, whereas 29.3% consumed breast milk and/or infant formula with water (mean = 58 g/day) and/or other beverages (mean = 115 g/day), such as 100% fruit juice, milk, and sugar-sweetened beverages (SSBs). For infants 6–11.9 months, appropriate beverages include breast milk, infant formula, and water; only 40.2% met these recommendations. Many 6–11.9 month olds consumed age-inappropriate beverages, including milk (31%) and SSBs (35%). After 12 months of age, appropriate beverages include water, milk, and a limited amount of 100% fruit juice and SSBs; 32.4% complied fully, 18.3% consumed appropriate and inappropriate beverages, and 49.3% consumed only inappropriate beverages. Among 12–23.9 month olds, 58% consumed milk, 18% juice, and 42% water while 63% consumed SSBs. Many infants and young children are not compliant with Mexican and international breastfeeding and complementary feeding guidelines for beverages. Communication and guidance about age-appropriate beverages should be improved.

## 1. Introduction

Human milk is the ideal nutrition source for infants. The World Health Organization recommends exclusive breastfeeding for the first six months of life and continuing frequent, on-demand breastfeeding until 2 years of age or beyond [1]. The Mexican Norm on Nutrition 043 is consistent with these recommendations, including gradual and progressive introduction of complementary foods from 6 months of age [2]. If a mother cannot or chooses not to breastfeed, fortified infant formula is an appropriate alternative [1].

While clear recommendations about infant feeding and complementary feeding exist [3], there is growing evidence that infants in Mexico are not fed according to recommendations. Breastfeeding rates in Mexico have declined in the last 10 years. According to the 2012 Mexican National Health and Nutrition Survey (ENSANUT, *Encuesta Nacional de Salud y Nutrición*), only 14.4% of Mexican babies are exclusively breastfed by 6 months of age [4].

Usual energy intakes of Mexican infants and toddlers (6–23.9 months) exceed estimated energy requirements in 14–32% of the population [5]. The extra energy has been linked to consumption of energy-dense foods low in micronutrients, including foods and beverages with added sugar and various maize-based preparations [6]. Sweetened tea and other sweetened beverages, including traditional non-milk beverages, are frequently consumed among infants and young children in Mexico [6,7] and may contribute to these excess energy intakes.

Approximately one-third of Mexican infants less than 1 year of age consume cow’s milk [6,7]. Cow’s milk is considered an inappropriate milk for children under 1 year, as early feeding of cow’s milk is associated with an increased risk of developing iron-deficiency anemia [8]. The reason behind this includes its low iron content, poor iron availability, and the associated occult intestinal blood loss [9].

Despite these various reports, no one has systematically compared intakes of milk and other beverages among infants and young children in Mexico to feeding guidelines and recommendations. The objective of this study is to evaluate recommendations for milk and other beverages by age, and examine consumption of age-appropriate milks and other beverages among Mexican infants and young children. We also explore socio-demographic associations among children adhering to beverage recommendations and those who do not to better understand the role that these beverages play in the diets of infants and young children in Mexico.

## 2. Methods

### 2.1. Study Population

The 2012 Mexican National Health and Nutrition Survey, ENSANUT, is a cross-sectional, probabilistic, population-based survey designed to characterize the health and nutritional status of the Mexican population. ENSANUT 2012 is a nationally representative study that used a multi-stage stratified sampling system drawn to be representative of four geographic regions in Mexico (North, Center, Mexico City, and South), by rural, urban, and socioeconomic strata [10]. Household demographic data were collected from the adult respondent and included education level, employment, marital status, socioeconomic status, and whether the child benefited from any type of food assistance program. The data were collected during October 2011 through May 2012 from 50,528 Mexican households with a household response rate of 87%. Dietary intake data were collected from a stratified, nationally representative subsample of participating households. We have included data from the 949 infants and young children up to two years of age for whom dietary intake data were available.

The survey protocol and data collection instruments were approved by the Ethics Committee of the Mexican National Institute of Public Health. Informed consent was obtained from each child’s parent/guardian for participation in the study [10].

### 2.2. Dietary Assessment

A single 24-h dietary recall was collected for each child by trained interviewers during a face-to-face interview with the parent or primary caregiver. Parents or caregivers reported detailed descriptions of all foods and beverages and the amounts consumed by the child for the previous 24-h period. To improve dietary recall data, ENSANUT 2012 implemented an automated five-step multiple-pass method [11,12] adapted to the Mexican context [13]. One 24-h dietary recall was completed for each of the participating children and could occur on either a weekday or a weekend day. A small subset completed a second 24-h recall [5]. The adaptations included translation to the Spanish language as well as modifications to reflect unique characteristics of food intake and preparation methods used in Mexico. The 24-h dietary recall data were also linked, for the first time, to a food and beverage composition table based on a compilation of the nutrient composition analyses conducted in Mexico and Central America (67% of foods), and the food composition tables from the USDA Nutrient Database for Dietary Studies [14] and the USDA National Nutrient Database for Standard Reference, release 26 [15] (33% of foods) [16]. Data on breastfeeding practices in children ages 0–35.9 months were based on an infant feeding practices questionnaire implemented on the day of the 24-h dietary recall, and breastfeeding amounts were estimated based on the child’s age and the total amounts of other milks consumed [5]. The total daily volume of breast milk was estimated to be 0.78 L/day for infants 0–5.9 months and 0.60 L/day for infants 6–11.9 months of age. If the child was partially breastfed, the volume of infant formula was subtracted from the estimated daily breast milk total. For those continuing to breast feed after 12 months, the volume assigned per feeding was 88.72 mL (3 fluid ounces) for children 12–17.9 months, and 59.15 mL (2 fluid ounces) for children 18–23.9 months old [5,17].

### 2.3. Beverage Groups

All milks and other beverages reported in the 24-h recalls were assigned to beverage groups. The list of beverage groups was based on the grouping system used in the U.S. Feeding Infants and Toddlers Study (FITS) [17,18]. Two trained Mexican dietary research specialists and a nutrition scientist at Nestlé modified these food groups and created additional groups to reflect the beverages commonly consumed by children in Mexico. Several Mexican beverage groups were added, including traditional beverages, such as *atoles* (hot beverages prepared with corn flour, milk, or water, sugar, and flavorings), *aguas frescas* (sweetened beverages prepared with fruit and water), *licuados* (smoothie-type drinks prepared with milk, fruit, and ice); and sweetened probiotic dairy drinks, such as Yakult^®^ [7].

Once the reported milks and other beverages were assigned to a beverage group, the groups were classified into seven mutually exclusive categories for analysis: breast milk, infant formula, water, 100% fruit juice, milk, sugar-sweetened beverages (SSBs), and artificially sweetened beverages [19]. Each category was then evaluated according to Mexican and international guidelines, and determined to be appropriate or inappropriate by age (Table 1).

Breast milk and infant formula are the only recommended milks for infants 0–5.9 months old [1,2,3,20]. From the age of 6 months, water is also allowed [2,20,21]. Beginning at the age of 12 months, 100% fruit juice [22] and whole cow’s milk [3,23] can be introduced. We considered that sweetened beverages should be limited due to the potential to over-consume and displace more healthful choices [3,23,24]. For the purposes of this research, sweetened beverages include both energy-dense, nutrient-poor beverages (such as sodas) as well as sweetened traditional beverages, such as *atoles* and *aguas frescas*, that may be made with milk or fruit juices.

### 2.4. Statistical Analysis

Analyses were based on the single 24-h dietary recall completed for all children. Consumers of the different beverages were defined as those who reported any consumption during the day of the recall, regardless of the amounts consumed. Stata version 14 (StataCorp, College Station, TX, USA) was used to create data files, assign individual beverages to beverage groups, and estimate the percentages of children who consumed beverages. For each beverage type, amount (g) per consumer and per capita were calculated. Daily energy (kcal) and amounts consumed from appropriate and inappropriate beverages were compared. ANOVA was used to compare demographic characteristics for the most commonly consumed inappropriate beverages by age and included water (0–5.9 months), SSBs and cow’s milk (6–11.9 months), and SSBs (12–23.9 months). A Bonferroni correction was applied for multiple comparisons. All estimates applied stratum-specific probability weights provided by ENSANUT to account for the complex survey design, resulting in nationally representative samples.

## 3. Results

**Figure 1:**
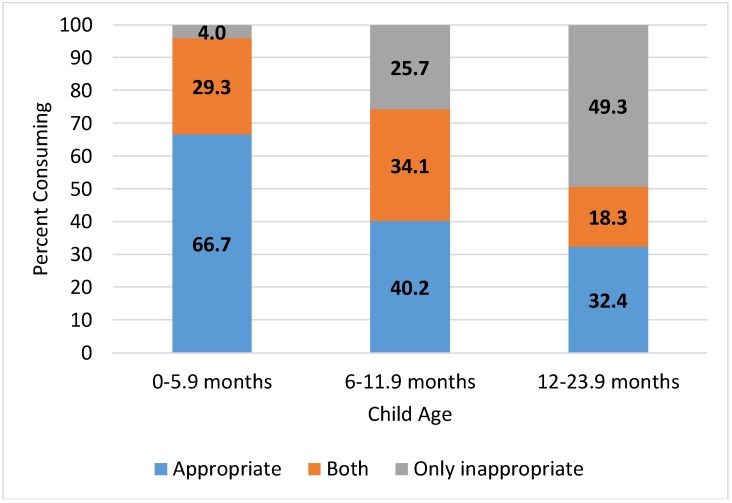
Percent of Mexican infants and toddlers consuming appropriate and inappropriate milk and beverages by age.

Adherence to beverage recommendations are summarized in Figure 1 and Table 2. Overall, approximately two-thirds of infants 0–5.9 months of age consumed only age-appropriate beverages, breast milk, or infant formula. Approximately 30% consumed breast milk or infant formula in addition to other age-inappropriate beverages, whereas 4% consumed only inappropriate beverages (no breast milk or infant formula) on the day of the survey. At 6–11.9 months of age, 40.2% consumed only appropriate beverages: breast milk, infant formula, or water. Approximately one-third consumed these appropriate beverages in addition to other age-inappropriate beverages, and one-quarter consumed only inappropriate beverages. From 12 months of age, only 32.4% consumed age-appropriate beverages while nearly half were consuming only sweetened beverages and no age-appropriate beverages at all. The amounts and energy contribution from appropriate beverages decreased with age, while inappropriate beverage consumption increased from 56 kcal/day among infants 0–5.9 months to approximately 200 kcal/d beginning at the age of 6 months (Table 2).

The consumption of appropriate and age-inappropriate beverages can be found in Table 3. The most commonly consumed inappropriate beverage among infants 0–5.9 months was water. However, among the few infants who consumed other inappropriate beverages, consumption was relatively high, ranging from 120 mL for juice to 500 mL for supplement drinks and 727 mL for sweetened milks. At 6 months, water becomes an appropriate beverage, and 35% of older infants reported water consumption. Inappropriate beverages for this age were most often milk (31%) and SSBs (35%). Average inappropriate milk consumption among consumers was 466 mL, close to two cups, and more than twice the amount of water consumed. In toddlers 12–23.9 months, appropriate beverages included water, 100% juice, and cow’s milk, which were consumed by 42%, 18%, and 58%, respectively. More children (63%) consumed SSBs than any other type of beverage, most commonly sweetened teas, infusions or coffee (23%), and traditional beverages (30%).

We selected the most commonly consumed inappropriate beverages by age, and compared various sociodemographic characteristics among those who consumed them and those who consumed only appropriate beverages (Table 4). Infants 0–5.9 months who consumed water tended to be older than four months and were more likely to consume breast milk compared to those who consumed only appropriate beverages. They were also less likely to come from the North of Mexico and more likely to be of normal weight. Older infants aged 6–11.9 months who consumed SSBs were more likely to be from households where the primary caregiver had less education. Inappropriate milk consumption in this age group was associated with lower consumption of breast milk or infant formula and occurred more frequently among urban dwellers. Inappropriate beverage consumption was also significantly higher among infants whose parent or caregiver was not employed. Among toddlers 12–23.9 months, SSB consumption was pervasive and did not differ in any meaningful way from those consuming appropriate beverages.

## 4. Discussion

This research provides a detailed picture of the consumption of breast milk, infant formula, water, and other beverages in the diets of infants and toddlers in Mexico. We compared consumption to Mexican and international policy and guidance documents to evaluate the proportions of these young children complying with recommendations or not and to characterize consumption of appropriate and inappropriate beverages by age.

In line with recommendations, 75% of infants from birth to 5.9 months of age consumed breast milk. More than half consumed infant formula alone or in combination with breast milk. However, only 67% of these young infants consumed only these appropriate beverages. A small study in the U.S. reported 53.1% of Latino infants at 4–6 weeks of age consuming infant formula, usually in combination with breast milk [25]. The rates of breastfeeding and infant formula consumption are also similar to that reported from the non-quantitative Infant and Young Child Feeding Practices questionnaire (IYCFP), a part of the ENSANUT 2012 [26], but whereas 19% of young infants were reported to consume water in our analysis of 24-h recall data, the IYCFP reported water consumption in infants less than 6 months at 51%. The study in Latino infants in the U.S. also confirmed a high percentage of infants consuming water at 4–6 weeks of age (56.3%) [25]. The differences may be due to the way data were collected, such as if water was separately coded from powdered infant formula or provided as a supplement.

The WHO/UNICEF Joint Monitoring Programme for Water Supply and Sanitation Water supplies indicates that 96.1% of Mexican households have access to “improved” water supplies [27], but this does not ensure microbiologically safe water [28]. For that reason, water used in the preparation of infant formula should be boiled for at least one minute and cooled before use [2,29]. Nevertheless, infants below the age of 6 months generally do not need water in addition to breast milk or properly prepared infant formula, even in warm climates [29,30]. It is for this reason that we classified water as an inappropriate beverage for young infants, but in practice, this should be evaluated on a case-by-case basis with the infant’s physician.

Other beverages were also consumed by young infants. We found that infants 0–5.9 months were consuming 100% juice (6%), milk (6%), and sugar-sweetened beverages or supplement drinks (8%) for a total of 20%, somewhat less than the 25.7% reported in the IYCFP [26]. Among those receiving milk and supplement drinks, the quantity (450–500 mL/day) would indicate that these were replacing age-appropriate breast milk or infant formula. Smaller amounts were consumed by infants reporting 100% juice, sweetened teas, herbal infusions or coffee, traditional beverages, and fruit-flavored drinks. Even so, these beverages replace necessary nutrition from breast milk or infant formula, potentially reducing the infant’s intake of micronutrients.

Breastfeeding was reported in 47% of older infants 6–11.9 months, comparable to 48% reported in IYCFP [26]. Fruit juice is not recommended for this age [22], but 13% consumed 100% fruit juice (116 mL/d), equivalent to about 0.5 cup (4 ounces) among consumers. Milk and SSBs are inappropriate at this age, but were consumed by 31% and 35%, respectively. As with younger infants, the amount of inappropriate milk consumed among consumers (about 470 mL; close to 2 cups per day), indicates that it was displacing breast milk and infant formula in this age group. One such milk was Liconsa, a fortified filled milk product provided at a reduced price through a federal program for families in need [31]. While we did not estimate intakes of Liconsa milk separately from other milks, the IYCFP reported 7% of infants 6–11.9 months were consuming this age-inappropriate milk [26]. Cow’s milk is inappropriate for infants as it increases risk for iron deficiency anemia in this vulnerable age group [8], and others have cautioned against the higher protein content of cow’s milk, increasing renal solute load and potentially increasing the risk for dehydration [32].

Consumption of SSBs was 63% in toddlers 12–23.9 months, an increase from 35% in older infants, consistent with that reported by Rodríguez-Ramírez et al. [6] but less than the 69.9% reported for non-nutritive liquids in IYCFP [26]. SSBs are not recommended at all for infants, and should be consumed with caution for toddlers as they provide excess energy and very few nutrients. Longitudinal studies in the U.S. and Portugal have shown that consumption of SSBs is associated with higher odds of obesity [33] and that consumption of energy-dense foods at age 2 years predicts consumption at age 4 years [34]. In Mexico, children in the highest tertile of cumulative consumption of SSBs from infancy to school-age were almost 3 times as likely to be obese at age 8–14 years [35].

Almost half of the SSBs consumed by toddlers are traditional Mexican beverages, and nearly one-quarter consume sweetened teas, herbal infusions, or coffee. Consumption is widely accepted, and many of the beverages are perceived to provide health benefits [3,36]. Parents may remember consuming these beverages themselves as children and consider them to be comfort foods. The Mexican Consensus on complementary feeding calls upon physicians and health professionals to educate themselves and guide parents away from frequent consumption of these beverages [3], largely because they provide excess calories with little nutritive value, increase dental caries risk, and for some beverages, provide caffeine.

We have found few other reports identifying demographic characteristics associated with adherence to feeding recommendations for milk and other beverages in infants and young children. National survey data on breastfeeding in the U.S. are not typically reported by ethnic subgroup [37]. However, U.S. breastfeeding rates were 49.5% in 2013 [38], similar to the 47% we observed for 6–11.9 month olds in the 2012 ENSANUT. In a study among 4–6 week old Latino infants in the U.S., 25.4% were fed water or teas, but early feeding of water and teas was not related to any maternal demographic characteristics [25]. We found that 20.9% of 0–5.9 month olds consumed water and that consumption was higher among breastfed children, while Wojcicki et al [25] found that tea and water consumption was higher among those consuming infant formula. This discrepancy could be due to the differences in age classifications, considering we also observed that water consumption was higher among infants older than 4 months.

A child’s perception of taste develops over infancy [39] and taste preferences are shaped over the first years of life. Although infants show an innate preference for sweet taste [40], early in complementary feeding, they will accept many foods and beverages, including very bland ones. One study found that 5–7 month-old infants showed positive reactions to 88% of foods tried [41]. Even foods and beverages that are not initially liked will often come to be accepted after the child tastes it repeatedly [42,43,44,45]. Although some have hypothesized that adding sweetness or energy to food could facilitate that process (flavor-flavor learning and flavor-nutrient learning, respectively), recent studies suggest that simple repeated exposure to the food, as-is, is just as effective [46,47].

Children’s preferences are shaped by the foods and beverages which they are offered in infancy. That is to say, children who are accustomed to consuming sweeter beverages will come to prefer them. One study showed that infants whose parents fed them sweetened water (e.g., with sugar or honey) at home consumed more sweetened water in the lab than infants who had not been exposed to sweetened water at home [48]; infants who had not received sweetened water at home consumed more plain water than sweetened. This can have long-term implications on later food preferences. Infants who had initially been fed with hydrolyzed or soy infant formulas, which tend to be more sour and bitter than standard milk-based infant formulas, showed better acceptance of broccoli at 4–5 years old [49]. For this reason, infant and young children’s early exposure to very sweet beverages could have long-term implications for their dietary preferences.

Our study is not without limitations. The ENSANUT 2012 is a cross-sectional survey, and while it comprehensively covers the country and provides national estimates of the population’s intakes, only one day of intake was reported. Habitual intakes may differ from this snap-shot view. The fact that breast milk volumes were estimated is also a limitation of this and other intake surveys of infants and young children [17,18,50]. We combined data from underweight and normal weight infants and young children in our analysis due to the small sample size for underweight children, but this does not necessarily mean that beverage consumption patterns would be the same. Another limitation is that taxes on sugar-sweetened beverages were introduced in Mexico in 2014, and this may have influenced purchase of SSBs, reducing intakes and increasing consumption of water [51]. We look forward to evaluating the longer-term effects of this policy change using data from the next Mexican national nutrition survey.

## 5. Conclusions

Breastfeeding rates of Mexican infants need to be improved to meet Mexican and international recommendations. Many infants and young children are not compliant with complementary feeding recommendations. Early consumption of cow’s milk before the age of 12 months may put some infants at risk for iron deficiency anemia. Even more problematic is the pervasive consumption of sugar-sweetened beverages, beginning in infancy and increasing to be the most frequently consumed beverages among toddlers. The Mexican consensus on infant feeding practices and other national and international recommendations are available to guide care-givers and parents on the proper feeding and timing of introduction of beverages complementary to breast milk and infant formula, but additional educational efforts are needed to improve feeding practices of infants and toddlers in Mexico.

## Figures and Tables

**Table 1:** Classification of milk and beverages as recommended or inappropriate for Mexican infants and toddlers by age.

Milks and Beverages	0–5.9 Months	6–11.9 Months	12–23.9 Months
Breast milk	The WHO, Mexican Norm on Nutrition 043, and the Mexican Consensus recommend exclusive breastfeeding until 6 months [1,2,3]; the AAP recommends ‘exclusive breastfeeding for about the first six months of a baby's life’ [20].	The WHO and Mexican Norm on Nutrition 043 recommend continued frequent, on-demand breastfeeding until at least 2 years of age [1,2]; the AAP recommends breastfeeding in combination with the introduction of complementary foods until at least 12 months of age [20].	The WHO and Mexican Norm on Nutrition 043 recommend continued frequent, on-demand breastfeeding until 2 years of age or beyond [1,2]; AAP recommends continued breastfeeding after 12 months for as long as mutually desired by mother and baby [20].
Infant formula			
Water		Mexican Norm on Nutrition 043 [2], AAP [20] and EFSA [21] indicate water can be introduced at 6 months.	Reference values for water intake include water from all sources: foods, beverages, and foods [21].
100% fruit juice			100% fruit juice can be a healthy part of the diet of children older than 1 year when consumed as part of a well-balanced diet [22]. ^b^
Milk			Full-fat cow's milk can be introduced at 1 year of age; reduced fat milk can be used after 2 years of age [23], or after 12 months if child weight gain is excessive. Other animal milks are acceptable, but milk substitutes (including plant milks) are not nutritionally equivalent to animal milk and are not recommended as a major food source [23].
Sugar-sweetened beverages (SSBs) ^a^			Excessive consumption of SSBs can contribute to energy imbalance [3,23,24]; traditional beverages are often high in sugar and should be limited as they can displace other healthier choices [3].
Artificially sweetened beverages			Mexican Consensus discourages artificially sweetened beverages for children under 2 years of age [3].

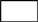
 White indicates Recommended beverages; 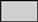 Dark grey indicates inappropriate beverages by age group; 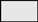 Light grey indicates beverages to limit due to sugar content. ^a^ Includes fruit-flavored drinks, carbonated sodas, sweetened tea and coffee (including herbal infusions), and Mexican traditional sweetened beverages. ^b^ Limited to at most 4 ounces (approximately 120 mL) per day for children ages 1–3 years of age. WHO = World Health Organization; AAP = American Academy of Pediatrics.

**Table 2:** Amounts (g) and energy (kcal) per capita provided by appropriate and inappropriate milk and beverages among Mexican infants and toddlers from ENSANUT 2012.

	Appropriate Beverage Consumption Only			Appropriate with Inappropriate Beverage Consumption		
	0–5.9 Months	6–11.9 Months	12–23.9 Months	0–5.9 Months	6–11.9 Months	12–23.9 Months
*n*	118	98	179	64	130	359
% consuming	66.7	40.2	32.4	33.3	59.8	67.6
Appropriate beverages, g (kcal)	1061 (648)	788 (476)	694 (329)	680 (444)	574 (220)	555 (225)
Water, g	NA	76	134	58	102	178
Inappropriate beverages, g (kcal)	NA	NA	NA	115 (56)	393 (227)	316 (193)

NA indicates not applicable.

**Table 3:** Consumption of appropriate and inappropriate milk and beverages among Mexican infants and toddlers in the ENSANUT 2012.

	0–5.9 Months (*n* = 182)			6–11.9 Months (*n* = 229)			12–23.9 Months (*n* = 538)		
	% ^a^	g/Consumer Mean ± SE	g/Capita Mean ± SE	% ^a^	g/Consumer Mean ± SE	g/Capita Mean ± SE	% ^a^	g/Consumer Mean ± SE	g/Capita Mean ± SE
Breast milk ^b^	75	669 ± 28	502 ± 35	47	536 ± 18	254 ± 26	14	286 ± 22	40 ± 7
Infant Formula	51	844 ± 201	429 ± 110	34	812 ± 167	273 ± 65	18	571 ± 66	101 ± 18
Water	19	100 ± 23	19 ± 6	35	223 ± 40	77 ± 19	42	291 ± 21	123 ± 12
100% fruit juice	6	120 ± 15	8 ± 3	13	116 ± 19	15 ± 4	18	181 ± 14	33 ± 6
Milk ^c^	6	456 ± 168	25 ± 12	31	466 ± 84	143 ± 30	58	432 ± 23	248 ± 18
Unsweetened ^d^	5	450 ± 171	24 ± 12	30	470 ± 88	139 ± 30	53	418 ± 22	221 ± 18
Sweetened ^e^	0	727 ± 0	1 ± 1	2	217 ± 65	4 ± 1	8	368 ± 83	28 ± 7
Sugar-sweetened beverages	8	99 ± 41	8 ± 4	35	187 ± 27	66 ± 12	63	282 ± 26	178 ± 19
Fruit-flavored drinks ^f^	1	81 ± 26	1 ± 1	9	226 ± 73	20 ± 9	15	266 ± 48	40 ± 11
Carbonated sodas	0	79 ± 71	0 ± 0	7	96 ± 18	6 ± 3	17	85 ± 10	14 ± 2
Sweetened tea, coffee ^g^	4	64 ± 17	2 ± 1	10	131 ± 27	13 ± 4	23	200 ± 22	46 ± 7
Traditional beverages ^h^	2	37 ± 5	1 ± 1	17	138 ± 35	23 ± 6	30	250 ± 35	75 ± 15
Supplement drinks ^i^	1	500 ± 0	4 ± 4	2	178 ± 55	4 ± 2	1	339 ± 85	4 ± 2
Artificially sweetened beverages ^j^	0	0 ± 0	0 ± 0	0	80 ± 0	0 ± 0	3	228 ± 44	6 ± 2

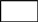
 White indicates appropriate beverages; 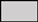 Dark grey indicates inappropriate beverages by age group; 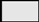 Light grey indicates beverages to limit due to sugar content. ^a^ Percent consuming (weighted); ^b^ Breast milk amounts were estimated and based on child age. ^c^ Includes cow’s milk and other milks (e.g., soy milk and other animal milks); % consuming other milks was 0 for 0–5.9 months, 1.8% for 6–11.9 months, and 1.8% for 12–23 month olds. ^d^ Includes plain unsweetened cow's milk and all other unsweetened milks. ^e^ Includes all sweetened cow’s milk and sweetened other milks (e.g., soy milk and other animal milks). ^f^ Fruit-flavored drinks containing <100% fruit juice and added sugar. ^g^ Sweetened tea, herbal infusions, and coffee. ^h^ Traditional sweetened beverages, including *atoles*, *licuados*, *aguas frescas*, and other beverages, such as Yakult^®^. ^i^ Fortified milks, such as Progresa^®^, and rehydration drinks, such as Gatorade^®^ and Suero^®^ (all of which contain or are served with sugar). ^j^ Artificially sweetened beverages were included in the total for SSBs as there were very few consumers. SE = standard error.

**Table 4:** Appropriate and inappropriate milk and beverage consumption among Mexican infants and toddlers by age and socio-demographic characteristics (ENSANUT 2012).

	0–5.9 Months (*n* = 182)		6–11.9 Months (*n* = 229)				12–23.9 Months (*n* = 538)	
Sociodemographic Characteristic	Appropriate Beverages (No Water) % Consuming	Inappropriate (Water) ^a^ % Consuming	Appropriate Beverages (No SSBs) % Consuming	Inappropriate (SSBs) ^b^ % Consuming	Appropriate Beverages (no Cow Milk) % Consuming	Inappropriate (Cow’s Milk) ^c^ % Consuming	Appropriate Beverages (No SSBs) % Consuming	Inappropriate (SSBs) ^d^ % Consuming
*n*	144	38	152	77	177	52	203	328
Mean child age (months)	3.3	4.2	9.0	9.9	9.1	10.0	18.2	18.2
Age <4 m	63	37 *						
Age ≥4 m	37	63 *						
Child sex								
Male	55	59	48	37	48	36	54	54
Female	45	41	52	63	52	64	46	46
Feeding mode								
Breast milk not infant formula	37	78 *	42	33	42	33	12	12
Infant formula not breast milk	24	9	32	13 *	36	0 *	23	13
Breast milk and infant formula	35	6 *	7	11	11	1 *	1	2
No breast milk/ infant formula		8		43		65 *	64	73
Region								
North	27	10 *	24	20	26	12	21	17
Central	35	29	31	40	32	41	34	27
South	33	33	32	37	40	19 *	30	37
Mexico City	6	28	13	3	2	28	15	19
Urbanicity								
Rural	29	22	24	29	30	16 *	31	31
Urban	71	78	76	71	70	84 *	69	69
Body weight								
Overweight/obese	18	2 *	8	22	11	17	6	9
Underweight/normal weight	82	98 *	92	78	89	83	94	91
Socio-economic status								
Lowest tertile	38	38	40	37	37	44	28	40
Middle tertile	30	44	33	41	38	30	48	28 *
Highest tertile	32	19	28	23	26	26	24	32
Dietary recall on weekday								
No	10	7	20	25	22	23	24	22
Yes	90	93	80	75	78	77	76	78
Child beneficiary of assistance program								
Food	7	16	5	5	4	6	4	7
Money	8	29	6	8	8	3	8	12
Medical	0	0	3	0	2	0	3	3
None	85	55	87	87	86	90	85	78
Education of primary caregiver								
Less than elementary	4	13	3	0 *	2	3	4	4
Elementary-secondary	86	82	86	98 *	90	91	84	84
More than high school	10	5	11	2 *	9	6	12	12
Primary caregiver employed								
No	68	79	78	71	70	89 *	67	69
Yes	32	21	22	29	30	11 *	33	31
Marital status of primary caregiver								
Separated or divorced, widowed, not married	21	30	16	23	16	24	16	12
Married, partners living together	79	70	84	77	84	76	84	88

^a^ Inappropriate beverages represented by water consumption (21% consuming); ^b^ Inappropriate beverages represented by SSB consumption (34% consuming); ^c^ Inappropriate beverages represented by cow’s milk consumption (23% consuming); ^d^ Inappropriate beverages represented by SSB consumption (62% consuming); * Significantly different from appropriate beverages group, *p* < 0.05; Bonferroni adjustment applied for multiple comparisons.

## Abbreviations

AAP	American Academy of Pediatrics
ENSANUT	Encuesta Nacional de Salud y Nutrición, the Mexican National Health and Nutrition Survey
IYCFP	Infant and Young Child Feeding Practices questionnaire
SSB	sugar-sweetened beverage
USDA	United States Department of Agriculture
